# Effect of SARS-CoV-2 on semen parameters: A meta-analysis of 39 articles from 15 countries

**DOI:** 10.7189/jogh.14.05021

**Published:** 2024-08-30

**Authors:** Lequan Wen, Haokun Tian, Xing Huang, Tiangang Song, Lirui Tang, Wenjie Wei, Shuo Tian, Yan Huang, Xu Zhang

**Affiliations:** 1 Department of Urology, The Third Medical Center, Chinese PLA General Hospital, Beijing, China; 2 Joint Program of Nanchang University and Queen Mary University of London, Nanchang University, Nanchang, China; 3 Nanchang Joint Programme, School of Biological and Behavioural Sciences, Queen Mary University of London, London, UK; 4 Department of Urology, First Affiliated Hospital of Nanchang University, Nanchang, Jiangxi, China

## Abstract

**Background:**

Declining birth rates during the pandemic have led to concerns about the potential impact of the of severe acute respiratory syndrome coronavirus 2 (SARS-CoV-2) on fertility among men. As previous studies have had inconsistent conclusions, we conducted a meta-analysis to evaluate the effects of SARS-CoV-2 on semen parameters.

**Methods:**

We searched several databases for articles published between 1 January 2020 and 25 July 2023. We performed a robust screening process based on predetermined inclusion and exclusion criteria and, following quality assessment, extracted data from high-quality studies for the meta-analysis. We determined the *P*-values and 95% confidence intervals (CIs) for both continuous and dichotomous variables, which we described using mean differences (MDs) and odds ratios (ORs), respectively. Lastly, we used the leave-one-out approach for our sensitivity analysis, and Begg’s and Egger’s tests to determine publication bias.

**Results:**

We included 39 articles with 1887 cases and 2097 controls. In patients infected with SARS-CoV-2, the sperm volume (MD = −0.29; 95% CI = −0.50, −0.07; *P* = 0.008) and concentration (MD = −8.71; 95% CI = −16.94, −0.48; *P* = 0.04) were decreased, which increased oligospermia risk (OR = 2.49; 95% CI = 1.04, 5.99; *P* = 0.04). Furthermore, we observed reduced sperm motility (MD = −8.18; 95% CI = −12.19, −4.17; *P* < 0.001) and increased immotility (MD = 4.06; 95% CI = 1.57, 6.54; *P* = 0.001) in infected patients, which increased asthenospermia risk (OR = 3.86; 95%CI = 1.83, 8.14; *P* = 0.0004). We also saw a decreased proportion of semen with normal sperm morphology (MD = −1.67; 95% CI = −2.68, −0.66; *P* = 0.001) and an increased proportion of semen with abnormal sperm morphology (MD = −1.31; 95% CI = −2.14, −0.49; *P* = 0.002,), along with increases in teratospermia (OR = 1.98; 95% CI = 1.00, 3.92; *P* = 0.05) in infected compared non-infected patients. Although we found consistency within most subgroups, we observed differences in severity, follow-up time, and country of origin. The results of the main meta-analysis results remained stable in the sensitivity analysis, while Begg’s and Egger’s tests showed no publication bias.

**Conclusions:**

Based on sufficient evidence, we see that the effects of SARS-CoV-2 on semen parameters resulted in a decline in male fertility. The increased severity and shorter duration of the SARS-CoV-2 infection increased the likelihood of altering of semen parameters.

**Registration:**

INPLASY: INPLASY202420083.

The severe acute respiratory syndrome coronavirus 2 (SARS-CoV-2), the virus causing the coronavirus disease 2019 (COVID-19), is characterised by high transmission rates and its significant impact on human health [1,2]. Once an individual is infected by the virus, he may experience a range of symptoms, including fever, cough, myalgia, fatigue, headache, haemoptysis, and diarrhoea [3,4]. Severe forms of COVID-19 can have drastic effects on their respiratory system, manifesting as pneumonia or acute aspiratory distress syndrome [5].

SARS-CoV-2, akin to the mumps virus [6] the Zika virus [7], can also negatively affect male reproductive function. A study conducted among 270 men and 189 women during the early pandemic, 37% of patients had decreased sexual activity and 44% had fewer sexual partners [8]. This was a consequence of social isolation or cardiovascular symptoms following COVID-19, which can contribute to erectile dysfunction and alter sexual desire and ejaculatory function [9]. In a study exploring the effects of COVID-19 on the male genital system, 1 patient had erythema, 8 had scrotal discomfort, 14 experienced swelling, and 16 experienced pain in the scrotal area; based on scrotal ultrasonography, 10 patients presented with acute orchitis, 7 with acute epididymitis, and 16 with acute epididymal orchitis [10]. In another study, patients with moderate COVID-19 have also been found to exhibit a significant reduction in sperm concentration in the semen, sperm motility, and the number of sperm per ejaculate, while those with mild COVID-19 did not significantly differ from controls in terms of semen parameters [11]. Although SARS-CoV-2 has been shown to affect the male genital system and decreases semen parameters [12], some studies have found that COVID-19 does not directly impair testicular function or semen parameters and that its indirect damage appears to be temporary [13]. The credibility of the results was limited because of the quality, sample size, and regional and ethnic disparities of the populations in the included studies. To provide reliable evidence for clinical practice, we aimed to conduct a meta-analysis of original studies on the impact of SARS-CoV-2 on parameters related to semen quality.

## METHODS

We registered the protocol for our study in the International Platform of Registered Systematic Review and Meta-analysis Protocols (INPLASY (INPLASY202420083)) after developing it based on PRISMA guidelines [14]. Two researchers independently retrieved the articles, screened them, assessed their quality, and extracted relevant data, discussing any disagreements in discussion with a third, external expert. We assessed the researchers’ inter-rater reliability using Cohen’s kappa coefficient.

### Search strategy

We searched PubMed, Embase, Web of Science, MedRxiv, BioRxiv, and the World Health Organisation Global Coronavirus databases using search terms such as sperm, semen, seminal fluid, spermatozoa, fertility, infertility, COVID-19, coronavirus disease 2019, SARS-CoV-2, SARS coronavirus 2, 2019-nCoV, and 2019 novel coronavirus (**Table 1**; Table S1 in the **Online Supplementary Document**). We limited our search to articles published between 1 January 2020 and 25 July 2023, without additional restrictions or filters.

**Table 1:** Retrieval strategy of PubMed

Query number	Query terms
#1	sperm
#2	semen
#3	seminal fluid
#4	spermatozoa
#5	fertility
#6	infertility
#7	#1 OR #2 OR #4 OR #5 OR #6
#8	COVID-19
#9	coronavirus disease 2019
#10	SARS-CoV-2
#11	SARS coronavirus 2
#12	2019-nCoV
#13	2019 novel coronavirus
#14	#8 OR #9 OR #10 OR #11 OR #12 OR #13
#15	#7 AND #14

### Inclusion criteria

We only considered English-language original research articles (regardless of study design) investigating the impact of SARS-CoV-2 on semen parameters in which the population was divided into case and control groups. To be eligible, these articles had to provide sufficient data on semen parameters, including those associated with sperm volume, concentration, motility, or morphology. During the follow-up of patients after SARS-CoV-2 infection, these data had to be available for the case group, regardless of their age, race, vaccination history, or differences in medication. We also considered the semen parameters of individuals not yet infected with SARS-CoV-2 in the control group for comparative analysis. We set no restrictions regarding the publication status of the articles.

### Exclusion criteria

We excluded studies without relevant data on the effects of SARS-CoV-2 on semen parameters; single-arm studies or those that only provided data on semen parameters at different follow-up time points after SARS-CoV-2 infection; or studies with incomplete data (for example, studies containing continuous variables, but only providing sample sizes, medians, and interquartile ranges). We also did not include articles published repeatedly by the same author and with smaller sample sizes, as well as review papers, meta-analyses, case reports, or animal studies.

### Quality assessment

We assessed the quality of the articles using the Newcastle-Ottawa Scale, excluding ones assessed as having poor quality from the meta-analysis.

### Data extraction

We extracted the name of the first author, year of publication, country, disease severity in the case group, follow-up status of the case group, and semen parameters from each article, as well as the number of samples in which the target event occurred and the total sample size for dichotomous variables, or the means and standard deviations and total sample size for continuous variables. We additionally extracted subgroup data on severity and follow-up status where applicable, except for studies where the subgroup classification was based on other factors (e.g. body temperature and medication), where we only extracted the overall data.

We classified the severity of COVID-19 as no symptoms, mild, moderate, severe, or combined/unclear, and follow-up as short, long, or combined/unclear. If the classification criteria for short- and long-term follow-up were not mentioned in the original article, we considered a follow-up of 90 days of symptom onset or confirmation of COVID-19 as short-term and a follow-up of 90 days or longer as long-term. We classified sperm motility status as either immotility or motility, with the latter being further characterised as progressive or non-progressive.

If multiple subgroups shared a single control group or if data on semen parameters were only provided on the subgroup rather than the overall level, we combined the data for each subgroup for the overall meta-analysis, as the control group could not be reused multiple times for the above-mentioned multiple subgroups. For dichotomous variables, we summed the number of samples in which the target event occurred and the total sample size for each subgroup. For continuous variables, we merged the data of each subgroup using the following formula:



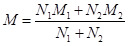




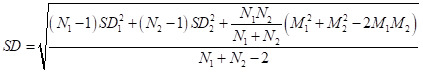


Here, *N*, *M*, and SD represent the sample size, mean, and standard deviation, respectively [15].

For continuous variables for which only the sample size, median, and first and third quantile, or only the sample size, median, Q0, and Q4 were provided, we estimated the mean and standard deviations using the quantile estimation (QE) method proposed by McGrath et al. [16,17]. For variables that could not be estimated using the QE method, we used the approach proposed by Wan et al. [18] and Luo et al. [19] were used [20]. For variables that could not be estimated using the QE method, we used the approach proposed by Luo et al. and Wan et al. was used [18-20].

### Statistical methods

We performed the meta-analysis in RevMan, version 5.4 (The Cochrane Collaboration, London, UK) and Stata, version MP 17 (StataCorp LLC, College Station, TX, USA). Per the Cochrane Handbook [21], we assessed for study heterogeneity using Cochran’s Q test. Per the Cochrane Handbook, we assessed for study heterogeneity using Cochran’s Q test, which followed a χ^2^ distribution with degrees of freedom equal to *k* − 1, where *k* is the number of studies. For our main meta-analysis, we used a fixed-effects model when heterogeneity was insignificant (*P* > 0.05) and a random-effects model when it was significant (*P* < 0.05). The fixed-effects model was based on all studies in the meta-analysis with a single true effect size. Sample errors accounted for any differences in the observed effects. The random-effects model assumes that because of study heterogeneity, the true effect may differ among studies [22]. We used a fixed-effects model when heterogeneity was insignificant (*P* > 0.05) and a random-effects model when it was significant (*P* < 0.05). If heterogeneity suggested *P* < 0.05, we conducted further subgroup analysis. However, we used fixed-effects models when fewer than five studies were included in the meta-analysis, irrespective of the results of the heterogeneity test [23].

We presented continuous and dichotomous variables as mean differences (MDs) and odds ratios (ORs), respectively. For the combined results meta-analysis, *P* < 0.05 indicated statistical significance. We did not perform a meta-analysis in cases where we had less than three studies for a certain parameter.

We otherwise performed our sensitivity analysis using the leave-one-out approach to determine if a single study affected the results of the meta-analysis, and we assessed for publication bias using Begg’s and Egger’s tests, with *P* > 0.05 indicating no significant publication bias.

## RESULTS

### Screening, quality assessment, and data extraction

**Figure 1:**
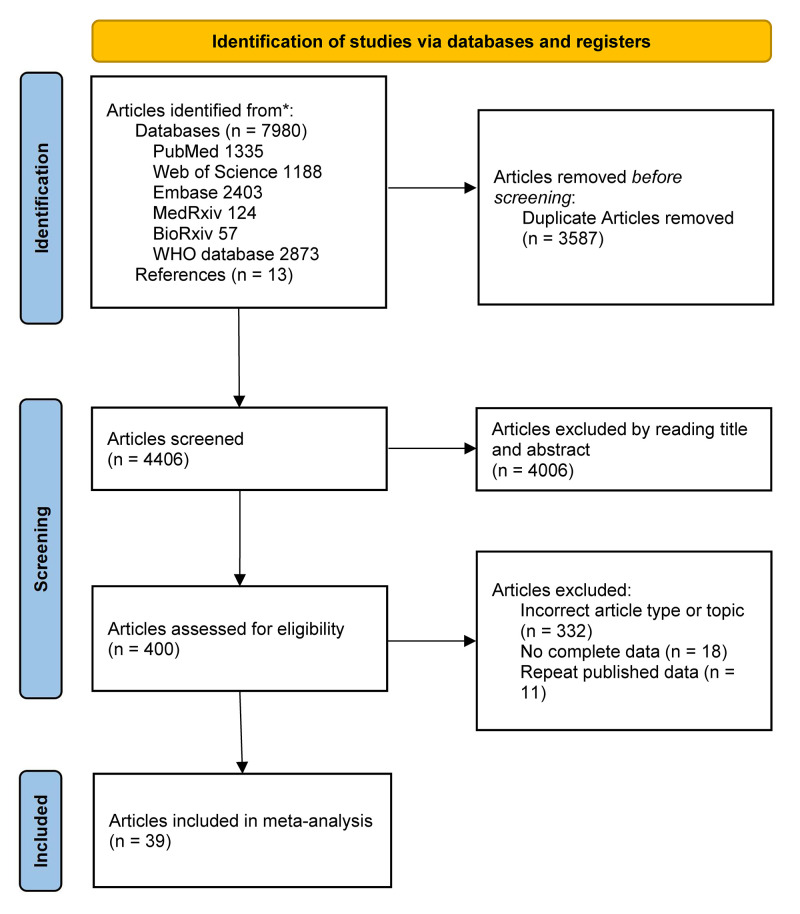
Flow diagram of article searching and screening.

After the title/abstract and full-text screening, we included 39 articles from 15 countries, encompassing 1887 cases and 2097 controls (**Figure 1**). The inter-rater agreement was categorised as excellent in the title/abstract (kappa = 0.846; 95% confidence interval (CI) = 0.819, 0.872) and full-text review (kappa = 0.928; 95% CI = 0.866, 0.991) stages. All the included articles were of adequate quality for the meta-analysis (Table S2 in the **Online Supplementary Document**). As the examined articles assessed the impairment of semen parameters in patients with COVID-19, the risk of reporting bias was considered low.

#### General information about the overall meta-analysis

We included 21 parameters in the meta-analysis. Continuous variables (presented as MDs) were sperm volume; sperm concentration; sperm vitality; sperm motility; sperm immotility; total sperm counts; motile sperm counts and progressively motile sperm counts, as well as progressive and non-progressive sperm motility; normal and abnormal sperm morphology; sperm head abnormal; sperm neck abnormal and sperm tail abnormal; pH; and DNA (DNA) fragmentation index. Dichotomous variables (presented as ORs) were oligospermia; asthenospermia; teratospermia; and the presence of white blood cells (WBC).

#### Meta-analysis of primary outcomes

The meta-analysis for the primary outcomes focussed on two general (sperm volume and concentration), two sperm motility statuses (sperm motility and immotility), and two sperm morphology parameters (normal and abnormal) (**Table 2**).

**Table 2:** Overall meta-analysis results

Variables	Number of studies	Heterogeneity	MD/OR (95 % CI)*	*P*-value
Sperm volume	34	Positive	−0.29 (−0.50, −0.07)	0.008
Sperm concentration	34	Positive	−8.71 (−16.94, −0.48)	0.04
Total sperm count	18	Positive	−32.50 (−82.73, 17.72)	0.20
Motile sperm count	5	Negative	−6.35 (−27.45, 14.76)	0.56
Progressively Motile sperm count	3	Negative	−10.60 (−32.02, 10.83)	0.33
Sperm vitality	6	Positive	−9.36 (−18.29, −0.44)	0.04
Sperm motility	23	Positive	−8.18 (−12.19, −4.17)	<0.001
Progressive sperm motility	31	Positive	−2.64 (−5.71, 0.43)	0.09
Non-progressive sperm motility	9	Negative	0.27 (−0.57, 1.11)	0.53
Sperm immotility	10	Negative	4.06 (1.57, 6.54)	0.001
Normal sperm morphology	18	Positive	−1.67 (−2.68, −0.66)	0.001
Abnormal sperm morphology	3	Positive	−1.31 (−2.14, −0.49)	0.002
Sperm head abnormal	3	Negative	1.13 (−0.02, 2.29)	0.054
Sperm neck abnormal	3	Negative	0.04 (−0.27, 0.36)	0.78
Sperm tail abnormal	3	Positive	0.05 (−0.47, 0.57)	0.85
pH	5	Negative	−0.02 (−0.07, 0.02)	0.28
DNA fragmentation index	4	Positive	4.01 (3.02, 5.00)	<0.001
Oligospermia	4	Negative	2.49 (1.04, 5.99)	0.04
Asthenospermia	3	Negative	3.86 (1.83, 8.14)	0.0004
Teratospermia	3	Negative	1.98 (1.00, 3.92)	0.049
WBC found	4	Negative	2.89 (1.30, 6.44)	0.009

CI – confidence interval, MD – mean difference, OR – odds ratio, WBC – white blood cell

*Continuous variables (presented as MD (95% CI) are sperm volume; sperm concentration; total sperm count; motile sperm count; progressively motile sperm count; sperm vitality; sperm motility; progressive sperm motility; non-progressive sperm motility; sperm immotility; normal sperm morphology; abnormal sperm morphology; sperm head abnormal; sperm neck abnormal; sperm tail abnormal; pH; and DNA fragmentation index. Dichotomous variables (presented as OR (95% CI)) are oligospermia; asthenospermia; teratospermia; and WBC found.

**Figure 2:**
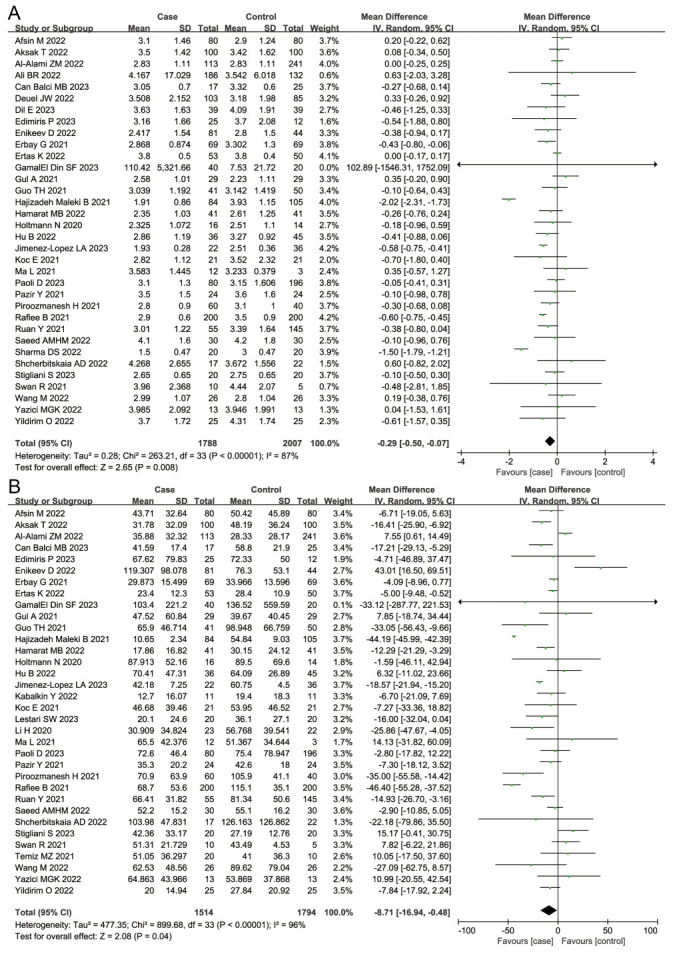
Overall meta-analysis results of sperm volume and sperm concentration. **Panel A.** Sperm volume. **Panel B.** Sperm concentration.

**Figure 3:**
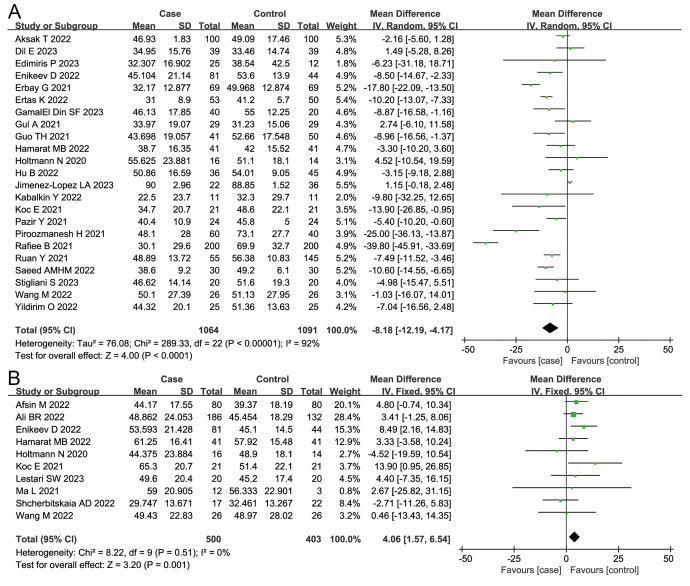
Overall meta-analysis results of sperm motility and sperm immotility. **Panel A.** Sperm motility. **Panel B.** Sperm immotility.

**Figure 4:**
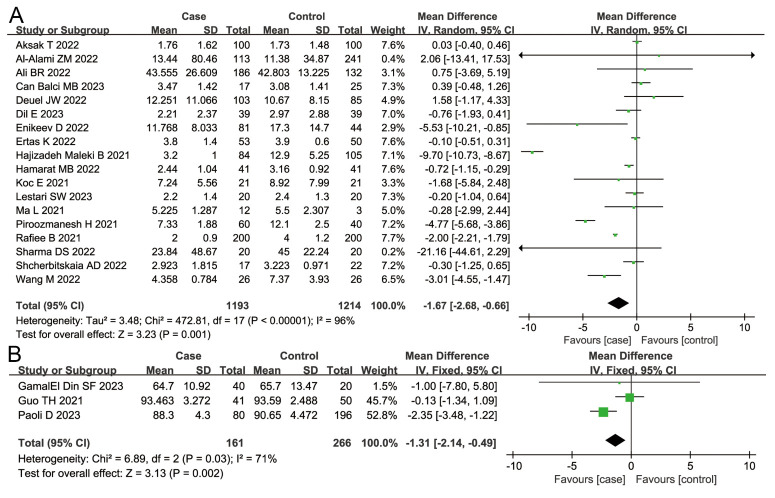
Overall meta-analysis results of normal sperm morphology and abnormal sperm morphology. **Panel A.** Normal sperm morphology. **Panel B.** Abnormal sperm morphology.

Considering the general parameters, we found that patients infected with SARS-CoV-2 had reduced sperm volume (MD = −0.29; 95%CI = −0.50, −0.07; *P* = 0.008) and concentration (MD = −8.71; 95% CI = −16.94, −0.48; *P* = 0.04) compared to those in the control group (**Figure 2**, Panels A and B). We also observed a decreased proportion of sperm motility (MD = −8.18; 95% CI = −12.19; −4.17; *P <* 0.001) and an increased proportion of sperm immotility (MD = 4.06; 95% CI = 1.57, 6.54; *P* = 0.001) in the case group when compared to the control group (**Figure 3**, Panels A and B), alongside a decreased proportion of normal sperm morphology (MD = −1.67; 95% CI = −2.68, −0.66; *P* = 0.001) and an increased proportion of abnormal sperm morphology (MD = 1.31; 95% CI = −2.14, −0.49; *P* = 0.002) (**Figure 4**, Panels A and B) .

#### Meta-analysis of secondary outcomes

Our meta-analysis of secondary outcomes looked at eight general parameters (total sperm count, sperm vitality, pH, DNA fragmentation index, oligospermia, asthenospermia, teratospermia, and presence of WBCs), four sperm motility status parameters (motile sperm count, progressively motile sperm count, progressive sperm motility, and nonprogressive sperm motility), and three sperm morphology parameters (abnormal sperm head, neck, and tail).

**Figure 5:**
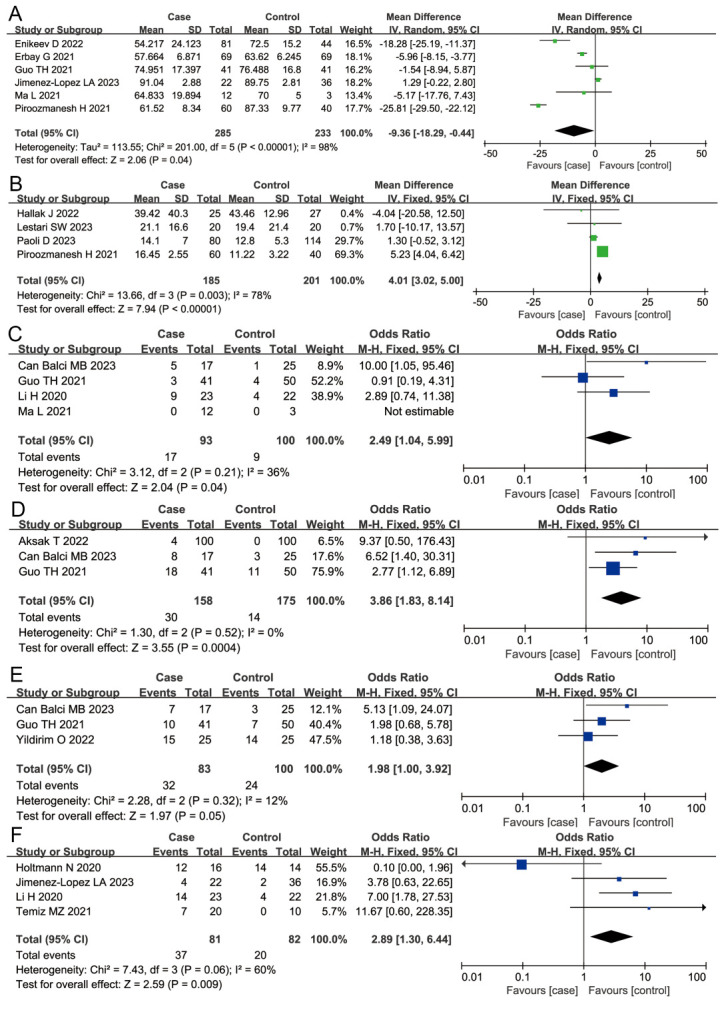
Overall meta-analysis results of sperm vitality, DNA fragmentation index, WBCs found, oligospermia, asthenospermia and teratospermia. **Panel A.** Sperm vitality. **Panel B.** DNA fragmentation index. **Panel C.** WBCs found. **Panel D.** Oligospermia. **Panel E.** Asthenospermia. **Panel F.** Teratospermia.

Here we observed that patients infected with SARS-CoV-2 had a reduced proportion of sperm vitality (MD = −9.36; 95% CI = −18.29, −0.44; *P* = 0.04) compared to the control group. The DNA fragmentation index was increased (MD = 4.01; 95% CI = 3.02, 5.00; *P* < 0.001) and WBCs were more likely to be present in the semen of COVID-19-infected patients (OR = 2.89; 95% CI = 1.30, 6.44; *P* = 0.009). The risk of oligospermia (OR = 2.49; 95% CI = 1.04, 5.99; *P* = 0.04), asthenospermia (OR = 3.86; 95% CI = 1.83, 8.14; *P* = 0.0004), and teratospermia (OR = 1.98; 95% CI = 1.00, 3.92; *P* = 0.05) was significantly higher after SARS-CoV-2 infection. However, we saw no statistical significance on the effect of SARS-CoV-2 on the total sperm count (MD = −32.50; 95% CI = −82.73, 17.72; *P* = 0.20) or pH (OR = −0.02; 95% CI = −0.07, 0.02; *P* = 0.28) (**Figure 5**, Panels A−E).

We saw no statistical significance regarding the effect of SARS-CoV-2 on motility (MD = −6.35; 95% CI = −27.45, 14.76; *P* = 0.56) and progressively motile sperm count (MD = −10.60; 95% CI = −32.02, 10.83; *P* = 0.33), or the proportion of progressive (MD = −2.64; 95% CI = −5.71, 0.43; *P* = 0.09) and non-progressive sperm motility (MD = 0.27; 95% CI = −0.57, 1.11; *P* = 0.53).

We also saw no significant difference in the effect of SARS-CoV-2 infection on the proportion of sperm head (MD = 1.13; 95% CI = −0.02, 2.29; *P* = 0.054), neck (MD = 0.04; 95% CI = −0.27, 0.36; *P* = 0.78), or tail (MD = 0.05; 95% CI = −0.47, 0.57; *P* = 0.85) abnormalities.

#### Subgroup analysis based on disease severity

In view of the subgroup analysis based on disease severity (**Table 3**), the effect sizes and statistically significant characteristics of most outcomes from the main meta-analysis remained consistent across the subgroups. However, there was a significant decrease in the proportion of progressive sperm motility in the mild subgroup (MD = −3.84; 95% CI = −5.67, −2.02; *P* < 0.001), while the reduction in sperm volume in the moderate subgroup was not statistically significant (MD = −0.14; 95%CI = −9.85, 4.22; *P* = 0.56).

**Table 3:** Subgroup analysis results based on severity

	Mild				Moderate				Severe				Combined/unclear			
**Variables**	**Number of studies**	**Heterogeneity**	MD/OR (95%CI)*	***P*-value**	**Number of studies**	**Heterogeneity**	MD/OR (95%CI)*	***P*-value**	**Number of studies**	**Heterogeneity**	MD/OR (95%CI)*	***P*-value**	**Number of studies**	**Heterogeneity**	MD/OR (95%CI)*	***P*-value**
Sperm volume	9	Negative	−0.53 (−0.67, −0.39)	<0.001	5	Positive	−0.14 (−0.64, 0.35)	0.56	3	Negative	−0.57 (−0.84, −0.30)	/	24	Positive	−0.28 (−0.56, 0.01)	0.06
Sperm concentration	10	Positive	−12.56 (−20.30, −4.81)	0.001	6	Negative	−6.80 (−12.65, −0.05)	0.02	/	/	/	/	/	Positive	−7.31 (−18.78, 4.16)	0.21
Total sperm count	6	Positive	−34.34 (−77.71, 9.04)	0.12	3	Negative	−25.91 (−50.50, −1.32)	0.04	3	Positive	−30.11 (−34.93, −25.29)	<0.001	12	Positive	−26.94 (−88.92, 35.04)	0.39
Motile sperm count	/	/	/	/	/	/	/	/	/	/	/	/	4	N	−7.53 (−29.23, 14.16)	0.50
Sperm vitality	3	Positive	0.50 (−0.89, 1.89)	0.48	3	N	−6.43 (−8.92, −3.94)	<0.001	/	/	/	/	/	/	/	/
Sperm motility	9	Positive	−6.21 (−11.40, −1.02)	0.02	4	P	−7.87 (−9.77, −5.98)	<0.001	/	/	/	/	14	Positive	−8.71 (−14.06, −3.36)	0.001
Progressive sperm motility	8	Negative	−3.84 (−5.67, −2.02)	<0.001	5	P	−2.82 (−9.85, 4.22)	0.43	/	/	/	/	22	Positive	−1.79 (−6.02, 2.44)	0.41
Non-progressive sperm motility	3	Negative	0.18 (−0.72, 1.09)	0.70	/	/	/	/	/	/	/	/	5	Negative	−0.59 (−1.85, 0.66)	0.35
Sperm immotility	3	Negative	0.11 (−4.32, 4.55)	0.96	/	/	/	/	/	/	/	/	6	Negative	4.16 (1.07, 7.25)	0.008
Normal sperm morphology	/	/	/	/	/	/	/	/	/	/	/	/	15	Positive	−1.81 (−2.90, −0.72)	0.001
Sperm head abnormal	/	/	/	/	/	/	/	/	/	/	/	/	3	Negative	1.13 (−0.02, 2.29)	0.054
Sperm neck abnormal	/	/	/	/	/	/	/	/	/	/	/	/	3	Negative	0.04 (−0.27, 0.36)	0.78
Sperm tail abnormal	/	/	/	/	/	/	/	/	/	/	/	/	3	Positive	0.05 (−0.47, 0.57)	0.85
pH	/	/	/	/	/	/	/	/	/	/	/	/	4	Positive	−0.04 (−0.99, 0.00)	0.07
DNA fragmentation index	/	/	/	/	/	/	/	/	/	/	/	/	4	Positive	4.01 (3.02, 5.00)	<0.001
WBC found	3	Positive	2.23 (0.85, 5.88)	0.11	/	/	/	/	/	/	/	/	/	/	/	/

CI – confidence interval, MD – mean difference, OR – odds ratio, WBC – white blood cell

c

In the mild subgroup, sperm vitality (MD = 0.50; 95% CI = −0.89, 1.89; *P* = 0.48), sperm immotility (MD = 0.11; 95% CI = −4.32, 4.55; *P* = 0.96), and the presence of WBCs (OR = 2.23; 95% CI = 0.85, 5.88; *P* = 0.11) did not differ significantly. The total sperm count, which did not exhibit statistical significance in the main meta-analysis, now saw a significant decrease among patients in the moderate (MD = −25.91; 95% CI = −50.50, −1.32; *P* = 0.04) and severe (MD = −30.11; 95% CI = −34.93, −25.29; *P* < 0.001) subgroups. These results suggest that the severity of semen damage may be related to the severity of the COVID-19 infection.

#### Subgroup analysis based on follow-up status

For the subgroup analysis based on the follow-up status (**Table 4**), we mostly saw consistency in terms effect sizes and statistical significance for the short-term follow-up subgroup with the overall meta-analysis. However, in the long-term follow-up subgroup, the previously observed differences in sperm volume (MD = −0.14; 95% CI = −0.55, 0.27; *P* = 0.50), proportion of sperm with normal morphology (MD = −0.06; 95% CI = −0.21, 0.10; *P* = 0.46), or sperm immotility (MD = 1.43; 95% CI = −3.04, 5.90; *P* = 0.53) were not statistically significant. As the duration of the post-COVID-19 infection follow-up increased, the extent of damage to a few semen parameters diminished. For instance, the impairment in sperm concentration was decreased in the short-term follow-up (MD = −14.31; 95% CI = −26.84, −1.77; *P* = 0.03) compared to the long-term follow-up subgroup (MD = −9.29; 95% CI = −15.86, −2.73; *P* = 0.006). Similarly, the damage to sperm vitality decreased in the short-term follow-up (MD = −12.01; 95% CI = −17.32, −6.70; *P* < 0.001) compared to that in the long-term follow-up subgroup (MD = −1.28; 95% CI = −2.50, −0.05; *P* = 0.04), as did the impairment in sperm motility (short-term follow-up subgroup: MD = −12.67; 95% CI = −20.39, −4.96; *P* = 0.001 vs long-term follow-up subgroup: MD = −6.80; 95% CI = −11.02, −2.58; *P* = 0.002). We saw a significant reduction in total sperm count in the long-term follow-up group (MD = −30.23; 95% CI = −54.39, −6.08; *P* = 0.01).

**Table 4:** Subgroup analysis results based on follow-up status

	Short-term follow up				Long-term follow up				Combined/unclear			
**Variables**	**Number of studies**	**Heterogeneity**	MD/OR (95%CI)*	***P*-value**	**Number of studies**	**Heterogeneity**	MD/OR (95%CI)*	***P*-value**	**Number of studies**	**Heterogeneity**	MD/OR (95%CI)*	***P*-value**
Sperm volume	15	Positive	−0.54 (−0.99, −0.09)	0.02	20	Positive	−0.14 (−0.55, 0.27)	0.50	10	Negative	−0.07 (−0.22, 0.08)	0.37
Sperm concentration	17	Positive	−14.31 (−26.84, −1.77)	0.03	18	Positive	−9.29 (−15.86, −2.73)	0.006	8	Positive	−7.88 (−17.18, 1.42)	0.10
Total sperm count	9	Positive	−58.63 (−138.34, 21.08)	0.15	9	Positive	−30.23 (−54.39, −6.08)	0.01	5	Negative	45292 (−6.65, 8.57)	0.79
Motile sperm count	/	/	/	/	/	/	/	/	3	Negative	−11.00 (−35.14, 13.13)	0.37
Sperm vitality	3	Positive	−12.01 (−17.32, −6.70)	<0.001	4	Negative	−1.28 (−2.50, −0.05)	0.04	/	/	/	/
Sperm motility	11	Positive	−12.67 (−20.39, −4.96)	0.001	15	Negative	−6.80 (−11.02, −2.58)	0.002	5	Positive	−8.84 (−17.85, 0.17)	0.054
Progressive sperm motility	14	Positive	−6.34 (−13.15, 0.48)	0.07	17	Positive	0.10 (−3.53, 3.74)	0.96	8	Negative	−2.79 (−4.36, −1.23)	0.0005
Non-progressive sperm motility	4	Negative	−0.58 (−2.73, 1.58)	0.60	3	Negative	0.22 (−0.82, 1.27)	0.67	3	Positive	0.41 (−1.25, 2.06)	0.63
Sperm immotility	6	Negative	7.05 (2.24, 11.87)	0.004	3	Negative	1.43 (−3.04, 5.90)	0.53	3	Negative	3.77 (0.31, 7.22)	0.03
Normal sperm morphology	10	Positive	−2.18 (−3.54, −0.82)	0.002	8	Negative	−0.06 (−0.21, 0.10)	0.46	7	Positive	−1.54 (−3.75, 0.68)	0.17
Abnormal sperm morphology	/	/	/	/	3	Negative	−2.11 (−3.15, −1.07)	<0.001	/	/	/	/
Sperm head abnormal	/	/	/	/	3	Negative	0.87 (−0.26, 1.99)	0.13	/	/	/	/
Sperm neck abnormal	/	/	/	/	3	Negative	0.21 (−0.15, 0.56)	0.25	/	/	/	/
Sperm tail abnormal	/	/	/	/	3	Negative	0.09 (−0.43, 0.61)	0.73	/	/	/	/
pH	4	Negative	0.04 (−0.02, 0.10)	0.18	3	Positive	−0.03 (−0.08, 0.02)	0.18	/	/	/	/
WBC found	4	Positive	2.71 (1.11, 6.62)	0.03	/	/	/	/	/	/	/	/

CI – confidence interval, MD – mean difference, OR – odds ratio, WBC – white blood cell

*Continuous variables (presented as MD (95% CI) are sperm volume; sperm concentration; total sperm count; motile sperm count; progressively motile sperm count; sperm vitality; sperm motility; progressive sperm motility; non-progressive sperm motility; sperm immotility; normal sperm morphology; abnormal sperm morphology; sperm head abnormal; sperm neck abnormal; sperm tail abnormal; pH; and DNA fragmentation index. Dichotomous variables (presented as OR (95% CI)) are oligospermia; asthenospermia; teratospermia; and WBC found.

#### Subgroup analysis based on the country

In our subgroup analysis by country (**Table 5**), the outcomes with statistically significant heterogeneity in the main analysis did not show significant heterogeneity in the Chinese subgroup. There was no statistically significant decrease in sperm volume of infected patients among patients from China (MD = −0.19; 95% CI = −0.43, 0.05; *P* = 0.12) and Turkey (MD = −0.07; 95% CI = −0.19, 0.05; *P* = 0.28). However, we noted a statistically significant reduction in the total sperm count in both Chinese (MD = −66.47; 95% CI = −98.08, −34.85; *P* < 0.001) and Turkish populations (MD = −22.18; 95% CI = −38.38, −5.97; *P* = 0.007). Lastly, we saw no significant difference exists in the risk of oligospermia (OR = 1.76; 95% CI = 0.65, 4.75; *P* = 0.27) among patients with COVID-19 in China, but we did note a decrease in the proportion of progressive sperm motility among infected patients in Turkey (MD = −4.17; 95% CI = −6.90, −1.44; *P* = 0.003).

**Table 5:** Subgroup analysis results based on country

	China				Iran				Turkey			
**Variables**	**Number of studies**	**Heterogeneity**	MD/OR (95% CI)*	***P*-value**	**Number of studies**	**Heterogeneity**	MD/OR (95% CI)*	***P*-value**	**Number of studies**	**Heterogeneity**	MD/OR (95% CI)*	***P*-value**
Sperm volume	5	Negative	−0.19 (−0.43, 0.05)	0.12	4	Positive	−0.84 (−0.96, 0.71)	<0.001	11	Negative	−0.07 (−0.19, 0.05)	0.28
Sperm concentration	6	Negative	−13.74 (−21.71, −5.76)	0.0007	4	Positive	−43.14 (−44.87, −41.41)	<0.001	11	Negative	−6.71 (−9.36, −4.07)	<0.001
Total sperm count	4	Negative	−66.47 (−98.08, −34.85)	<0.001	/	/	/	/	7	Negative	−22.18 (−38.38, −5.97)	0.007
Sperm motility	4	Negative	−6.39 (−9.39, −3.38)	<0.001	3	Positive	−29.31 (−33.98, −24.65)	<0.001	8	P	−6.10 (−10.96, −1.24)	0.01
Progressive sperm motility	5	Negative	0.15 (−2.59, 2.88)	0.92	/	/	/	/	10	P	−4.17 (−6.90, −1.44)	0.003
Non-progressive sperm motility	/	/	/	/	/	/	/	/	3	Negative	−1.09 (−2.95, 0.78)	0.25
Sperm immotility	/	/	/	/	/	/	/	/	3	Negative	5.19 (1.09, 9.29)	0.01
Normal sperm morphology	/	/	/	/	3	Positive	−2.42 (−2.62, −2.22)	<0.001	6	Negative	−0.24 (−0.47, −0.01)	0.04
Oligospermia	3	Negative	1.76 (0.65, 4.75)	0.27	/	/	/	/	/	/	/	/

CI – confidence interval, MD – mean difference, OR – odds ratio, WBC – white blood cell

*Continuous variables (presented as MD (95% CI) are sperm volume; sperm concentration; total sperm count; sperm motility; progressive sperm motility; non-progressive sperm motility; sperm immotility; and normal sperm morphology. Oligospermia is treated as a dichotomous variables (presented as OR (95% CI)).

#### Sensitivity analysis

For the sperm volume parameter, the MD values were less than zero and the *P*-values were less than 0.05 after the exclusion of any single study, suggesting strong stability. We also saw greater stability for parameters such as sperm motility. However, for sperm concentration, total sperm count, sperm vitality, progressive sperm motility, DNA fragmentation index, and the presence of WBC, which were limited by factors such as heterogeneity and the number of included studies, we observed changes in statistical significance after the exclusion of any specific, single study.

#### Publication bias

**Figure 6:**
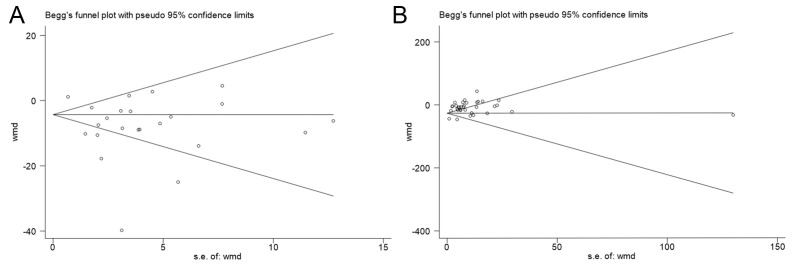
Funnel plots of sperm motility and sperm concentration. **Panel A.** Sperm motility. **Panel B.** Sperm concentration.

While all studies that looked at sperm motility were distributed almost symmetrically along the axis of symmetry, suggesting a low risk of publication bias, we did not observe this symmetry for sperm concentration, suggesting possible publication bias (**Figure 6**, Panels A and B). The results of the quantitative Begg’s and Egger’s tests indicated that none of the parameters showed significant publication bias except for sperm concentration, total sperm count, and sperm motility *(P <* .05) (**Table 6**). Therefore, except for these three parameters, the results of the meta-analysis for most of the parameters were less affected by publication bias, meaning that its results are highly reliable.

**Table 6:** Overall publication bias analysis results based on Begg’s and Egger’s tests

	Begg’s test		Egger’s test	
**Variables**	**Z**	***P*-value**	**t**	***P*-value**
Sperm volume	0.42	0.678	1.19	0.243
Sperm concentration	2.91	0.004	3.76	0.001
Total sperm count	1.97	0.049	0.60	0.560
Motile sperm count	−0.24	1.000	0.87	0.449
Progressively Motile sperm count	0.00	1.000	-0.36	0.778
Sperm vitality	0.00	1.000	−1.19	0.301
Sperm motility	0.74	0.460	−2.15	0.043
Progressive sperm motility	0.27	0.786	0.45	0.653
Non-progressive sperm motility	0.10	0.917	0.03	0.976
Sperm immotility	0.36	0.721	−0.42	0.688
Normal sperm morphology	1.74	0.081	−0.18	0.858
Abnormal sperm morphology	0.00	1.000	0.10	0.938
Sperm head abnormal	1.04	0.296	−2.74	0.223
Sperm neck abnormal	0.00	1.000	0.64	0.638
Sperm tail abnormal	0.00	1.000	−0.30	0.815
pH	−0.24	1.000	0.29	0.793
DNA fragmentation index	0.34	0.734	−0.74	0.534
Oligospermia	0.00	1.000	0.73	0.601
Asthenospermia	0.00	1.000	2.07	0.286
Teratospermia	0.00	1.000	1.52	0.370
WBC found	1.02	0.308	−0.95	0.444

WBC – white blood cell

## DISCUSSION

Our study is one of the first meta-analyses to evaluate the changes in semen parameters after SARS-CoV-2 infection. We included the 21 most commonly used parameters for the evaluation of male fertility, such as sperm volume, concentration, motility, immotility, and morphology, extracting them from 39 high-quality articles from 15 countries.

Our results indicate that SARS-CoV-2 infection is associated with altered semen parameters. This infection correlated with decreased sperm volume and concentration, resulting in a significantly higher risk of oligospermia (*P* < 0.05). We also observed a decreased proportion of semen with normal sperm motility and morphology and an increased proportion of semen with sperm immotility and abnormal sperm morphology in COVID-19-infected patients, resulting in an increased risk of asthenospermia and teratospermia (*P* < 0.05). We further observed reduced sperm vitality in these patients. In semen samples from men infected with SARS-CoV-2, the probability of WBC presence and increased DNA fragmentation index were significantly greater compared to that in the non-infected individuals (*P* < 0.05).

The effect sizes and statistical significance from the main meta-analysis remained consistent in most subgroup analyses. These findings indicate that the severity of the SARS-CoV-2 infection is associated with impaired semen parameters. Specifically, severe infection is more likely to affect patient semen parameters, as supported by the results on the total sperm count. Furthermore, previous studies have shown that, compared to mild SARS-CoV-2 infection, severe infection is more likely to be linked with intense immune and inflammatory reactions and damage to the urinary system [24], as well as disorders of the male reproductive system [25]. Although many studies showed similar trends, the progressive sperm motility parameter presented different trends in our meta-analysis, which could represent random events from the small number of included studies.

The follow-up duration affected certain semen parameters. We observed that SARS-CoV-2 infection was associated with a significant decrease in sperm volume, a decrease in the proportion of semen with normal sperm morphology, and an increase in sperm immotility in the group with short-term follow-up. However, these parameters seemed to largely recover with increasing follow-up time. Other studies have reported a temporary relationship between SARS-CoV-2 infection and certain semen parameters [13]. Although many parameters in our analysis conformed to this pattern, sperm concentration, vitality, and motility did not completely recover over time. However, as we previously mentioned, these parameters recovered to varying degrees when extending follow-up time. Furthermore, the impact of SARS-CoV-2 infection on semen may be chronic and persistent [26]; however, further research is required to confirm these findings.

Moreover, our subgroup analysis indicated that certain semen parameters after SARS-CoV-2 infection varied between populations from different countries. Owing to limited original data, we only compared the differences among studies from China, Iran, and Turkey. We observed no significant difference in the total sperm count due to SARS-CoV-2 infection in the overall analysis; however, in the subgroup analysis, we found a statistical difference between China and Turkey. The overall analysis showed that SARS-CoV-2 infection was significantly associated with sperm volume, leading to an increased risk of oligospermia. However, we saw significant changes in semen parameters in Chinese patients in the subgroup analysis.

The consistency of these results suggests an association between viral infection and male fertility potential, while the differences in subgroup analyses highlight the impact of severity, follow-up duration, and race on specific semen parameters. Although we detected heterogeneity in certain pooled results, this was to be expected, as the studies were conducted in different periods and adhered to different diagnostic criteria, and technical parameters between study centres [27]. For studies showing significant heterogeneity, the exclusion of individual studies may render the heterogeneity non-significant. For instance, the exclusion of Erbay and colleagues’ [28] multicentre prospective cohort study led to a decrease in the heterogeneity of semen volume in the subgroup with severe COVID-19, as indicated by a change in the *P*-value of Cochran’s Q test from 0.01 to 0.12. Similar results were observed in a related meta-analysis [29]. Previous meta-analyses suggested that factors such as the globalisation of data sources, variations in study protocols, and differences in sampling techniques significantly contribute to heterogeneity [30,31].

Here we summarised the available literature on the effect of SARS-CoV-2 infection on semen parameters over a specific period (2020–23). Based on the criteria of the 2010 World Health Organization Laboratory Manual for the Examination and Processing of Human Semen [32], we observed that SARS-CoV-2 infection can be linked to a significant decrease in semen volume and concentration, but that these decreases do not extend below the physiological minimum. However, we observed that SARS-CoV-2 infection can be associated with abnormal levels of sperm motility and abnormal sperm morphology in most patients, and these values are not within the physiological limits. The invasion of the virus into the reproductive system of human males cannot be completely blocked by the blood-testis barrier [33]. To date, many viruses that induce orchitis and male infertility have been detected in human semen samples, including the Marburg [34], Zika [35], and Ebola [36] viruses. SARS-CoV-2 can be detected in the urine of men [37], indicating virus invasion of the male genitourinary system. Testicular pain is an important symptom of SARS-CoV-2 infection in young men [38,39], while the mechanism by which the virus affects the male reproductive system remains unclear. Currently, only a few studies found residual SARS-CoV-2 RNA in semen [40], suggesting that SARS-CoV-2 possibly cause damage to the semen parameters of infected patients through the indirect effect of the virus on the reproductive system [41]. The long fever duration caused by COVID-19 may interrupt the cell cycle, affecting the protection of heat shock proteins against high body temperature [42]. Additionally, the immune system responses triggered by viral infection, including hemophagocytic lymphohistiocytosis [43]; interferon release caused by the infiltration of leukocytes, CD68+ macrophages, and CD3+ T lymphocytes into the testicular interstitial tissue [44–46]; and increased plasma levels of cytokines [3,47] may inhibit the male reproductive system, harming semen parameters. Moreover, excessively high levels of reactive oxygen species negatively affect proteins and lipids in the plasma membrane of the sperm and cause sperm DNA to break down [48]. At the molecular level, ACE2, the entry point into cells for SARS-CoV-2, is highly expressed in the male genital tract and human testis [49]. This is predominantly observed in spermatozoa, Leydig cells, and Sertoli cells [50,51]. Through ACE2, SARS-CoV-2 enters cells via its surface S proteins and can induce or inhibit autophagy by itself, or it may depend on ACE2 to promote viral survival. This process could partially explain the mechanism of SARS-CoV-2-induced male reproductive disorders [52]. However, most of the studies in our analysis were observational, so we cannot draw inferences regarding this mechanism.

This study has certain limitations. First, we only included published English-language articles and preprints in our analysis, omitting grey articles or those published in other language. Second, the original studies differed in their definitions of short- and long-term follow-up, such as a two- or three-month threshold, with some not providing a clear definition. Therefore, we defined short- and long-term follow-up subgroups based on the original articles and our own classifications. We also could not conduct further subdivisions or explore the specific duration of recovery due to the small number of studies in each subgroup. Further, the limited data on progressively motile sperm count, pH, DNA fragmentation index, oligospermia, asthenospermia, teratospermia, presence of WBC, and most sperm morphology parameters meant we could only include a small number of studies in the meta-analysis and a small number of case and control groups, reducing the power of our conclusions. Moreover, while patient age is an important factor affecting semen quality, we were unable to conduct a subgroup analysis because most included studies did not distinguish between age groups; this reduced the comprehensiveness of our results. Furthermore, separate analyses on participants with factors that could potentially affect semen parameters (such as lifestyle, illnesses, or medication history) were performed in a limited number of studies, so we could not include data for this specific category and conduct additional analyses. Finally, because our meta-analysis was mainly based on observational studies, further research is necessary to validate the association between SARS-CoV-2 infection and semen parameters. For example, longitudinal studies with larger sample sizes could offer more insight into the timing of these effects and help clarify the relationship between the severity and duration of SARS-CoV-2 infection and male reproductive health. Studies that distinguish subgroups based on factors such as age and medical history are also needed, as is research on potential interventions to mitigate COVID-19's effects on semen quality is crucial for the development of targeted therapeutic strategies.

The results of this meta-analysis have important implications for clinical practice and public health policies. Healthcare providers should be aware of the potential effects of SARS-CoV-2 on semen quality, particularly in male patients with severe infection and during the period shortly after the onset of infection. Furthermore, it may be necessary to provide reproductive health advice to individuals who recover from infection, so regular assessment of semen parameters should be made a part of post-COVID-19 care. Fertility counselling may be applied to those planning to have children through early intervention and customised medical plans to alleviate the long-term adverse effects of the epidemic on reproductive health. From a public health policy perspective, the observed impact on semen parameters underscores the need for ongoing monitoring and research into the long-term consequences of SARS-CoV-2 infection, thus, developing strategies for fertility preservation. Policymakers should consider including reproductive health evaluations in post-COVID-19 care to ensure that individuals are informed about the potential risks. Furthermore, public health campaigns should raise awareness about the potential effects of SARS-CoV-2 on male fertility.

## CONCLUSIONS

In this meta-analysis based on 39 studies, we saw decreases in the sperm volume and concentration of patients infected with SARS-CoV-2, causing an increased risk of oligospermia. We also observed decreases in sperm motility and increases in sperm immotility in this population, resulting in an increased risk of asthenospermia. Regarding sperm morphology, the proportion of normal sperm morphology was reduced in patients with SARS-CoV-2, while the proportion of abnormal sperm morphology increased with an increased risk of teratospermia. We also found that SARS-CoV-2 infection limits sperm vitality, while SARS-CoV-2-infected patients had increased DNA fragmentation and the presence of WBC in the semen. In addition, differences in a few parameters, such as the total sperm count and progressive sperm motility, were statistically significant in certain subgroups. For example, the results of our subgroup analysis showed that the total sperm count was significantly affected by disease severity and ethnic differences, and that parameters such as sperm volume, immotility, and proportion of semen with normal morphology changed significantly with follow-up time. A higher severity or shorter duration of SARS-CoV-2 infection increased the likelihood of semen parameters being affected. The data we used in our meta-analysis came from 15 developing and developed countries on different continents, as well as from groups such as Caucasians, Mongolians, and Black people. This makes our results representative of diverse populations and contexts. Although sensitivity and publication bias analyses showed that the conclusions of our meta-analysis are relatively stable and that its robustness is not easily affected by publication bias, additional large, multicentre, high-quality studies are warranted to provide more evidence for clinical practice.

## Additional material


Online Supplementary Document
